# Changes in the Global Epidemiology of Type 1 Diabetes in an Evolving Landscape of Environmental Factors: Causes, Challenges, and Opportunities

**DOI:** 10.3390/medicina59040668

**Published:** 2023-03-28

**Authors:** Ioannis Ogrotis, Theocharis Koufakis, Kalliopi Kotsa

**Affiliations:** 1 School of Medicine, Faculty of Health Sciences, Aristotle University of Thessaloniki, 54124 Thessaloniki, Greece; 2 Division of Endocrinology and Metabolism and Diabetes Center, First Department of Internal Medicine, Medical School, Aristotle University of Thessaloniki, AHEPA University Hospital, 54636 Thessaloniki, Greece

**Keywords:** type 1 diabetes, autoimmunity, epidemiology, environmental factors

## Abstract

The worldwide incidence of type 1 diabetes mellitus (T1DM) has increased in recent decades. The reasons behind this phenomenon are not yet fully understood. Early life infections, prenatal and perinatal factors, and diet composition have been associated with the triggering of autoimmunity and the risk of presentation of T1DM. However, the rapid increase in new cases of the disease raises the hypothesis that lifestyle factors, which have traditionally been associated with type 2 diabetes, such as obesity and unhealthy eating patterns could also play a role in the genesis of autoimmune diabetes. This article aims to highlight the changing epidemiology of T1DM and the importance of properly recognizing the environmental factors behind it, as well as the connections with the pathogenesis of the disorder and the need to prevent or delay T1DM and its long-term complications.

## 1. Introduction

In 2021, the number of individuals living with T1DM was estimated to be approximately 8.4 million worldwide, with 500,000 new cases that year. By 2040, the number of people living with T1DM is projected to reach 13.5–17.4 million. The life expectancy of a 10-year-old child diagnosed with T1DM is on average only 13 years in low-income countries and 65 years in high-income countries [1]. Significant complications, including cardiac diastolic dysfunction, dyslipidemia, and albuminuria, have been reported in adolescents with T1DM, and endothelial dysfunction has been reported even earlier during childhood [2]. Furthermore, approximately 30% of patients with T1DM develop end-stage kidney disease [3] during their lifetime. Poor quality of life and psychological burden are also associated with T1DM [4,5]. Therefore, it is becoming evident that the increase in the incidence of T1DM worldwide poses a serious challenge to public health.

The prevalence of T1DM increases annually by 0.34% [6]. The contribution of environmental factors to the initiation of the autoimmune process that results in the destruction of β-cells has been thoroughly investigated. Perinatal parameters, such as mode of delivery, and maternal and childhood diet, and ecological contributors, such as atmospheric pollution and climatic conditions, have also been hypothesized to correlate with the prevalence of T1DM. The epidemic of obesity, which leads to insulin resistance and lipid disorders, has been suggested to accelerate the presentation of T1DM in genetically predisposed individuals [7]. This article aims to highlight the changing epidemiology of T1DM and the importance of properly recognizing the environmental factors behind it, as well as the connections with the pathogenesis of the disorder and the need to prevent or delay T1DM and its long-term complications.

## 2. Rationale and Search Strategy

The growing prevalence of T1DM cannot be attributed to genetic reasons; therefore, possible explanations should be sought in the changing landscape of environmental factors, given that societies and human lifestyles tend to evolve over time. Among the multitude of environmental contributors involved in the pathophysiology of T1DM, this article will focus on those related to the increasing prevalence of the disease worldwide. The databases PubMed, Embase, and Google Scholar were searched to identify relevant articles written in the English language, regardless of the type of article or year of publication. A combination of the following search terms was used: “type 1 diabetes mellitus”, “autoimmune diabetes”, “prevalence”, “incidence”, “epidemiology”, “environment”. The references of all relevant studies and reviews were scanned for additional articles. The last search was performed in February 2023.

## 3. Lifestyle Factors

Traditionally linked to type 2 diabetes mellitus (T2DM), lifestyle factors have recently been associated with T1DM. The parallel epidemic increase in the incidence of T1DM and T2DM raises the hypothesis that this could be attributed to common causes [8]. A high body mass index (BMI) has been associated with an earlier onset of T1DM [9,10,11,12], although some studies produced conflicting results [13,14]. In fact, there appears to be a significant correlation between mean BMI and incidence of T1DM [15]. Furthermore, a higher BMI during childhood comprises an independent risk factor for the development of T1DM later in life [16,17].

Cholesterol levels are an additional factor related to lifestyle and Τ1DM. The incidence of T1DM has been associated with the average cholesterol concentrations of the population of a country [18]. Furthermore, statins have been shown in both human studies and animal models to help preserve β-cell function [19,20]. The pathways through which hypercholesterolemia predisposes individuals to T1DM are by inducing the Th1 response [21], oxidative stress, promoting β-cell death [22], and apoptosis [23]. Finally, the incidence of T1DM has also been associated with the mean consumption of meat in a country, and a diet rich in proteins and fermentable fibers [24,25,26]. Therefore, modern societies with an abundance of goods and easy access to them are expected to have a higher incidence of T1DM [27]. A well-documented paradigm of this phenomenon occurred after the reunification of Germany, in Saxony, where the incidence of T1DM increased drastically [28].

These observations gave birth to the accelerator hypothesis. The hypothesis, first published in 2001 [29], argues that both T1DM and T2DM are disorders related to insulin resistance that occur in patients with different genetic backgrounds [30]. Therefore, it questions the primacy of autoimmunity in the causation of T1DM. Instead, insulin resistance triggers the cataract of events that finally leads to the autoimmune destruction of hyperfunctioning β-cells, as a result of glucotoxicity [31]. Several mechanisms have been proposed through which increased adiposity promotes the development of T1DM. It is well established that the obese state is associated with low-grade inflammation and the release of pro-inflammatory cytokines secreted by macrophages that penetrate adipose tissue. These cells contribute to the presentation of autoantigens by islet cells, setting the stage for the vicious circle of autoimmune disorder that characterizes T1DM [7]. In addition, insulin resistance as a result of excessive adiposity has been associated with the formation of neo-epitope antigens, the acceleration of β-cell apoptosis and a rapid decrease in insulin secretory capacity [9,10].

Although it is reasonable to assume that diabetes will develop earlier in an insulin-insensitive individual compared to a sensitive one, and that accelerators are undoubtedly present in this process, the accelerator hypothesis has been debated [32]. Blom et al. showed that although children of both sexes with T1DM were significantly higher compared to normoglycemic controls, the weight-to-height ratios were similar, suggesting that linear growth, but not obesity, was associated with T1DM [33]. Recently, 91.6% of new cases of autoimmune diabetes during childhood were shown to have normal weight at the time of diagnosis [34]. On the other hand, a study conducted in the Finnish population indicated that a relative weight >120% after 3 years of age is associated with a more than double increase in the risk of T1DM [12]. Although the association between increased BMI and autoimmune diabetes appears to be there, it should be noted that the absolute differences in weight between children with T1DM and controls are actually small. In the study by Knerr et al. patients diagnosed between 15 and 20 years had a mean BMI of 21.6 kg/m^2^ compared to 20.6 kg/m^2^ of the reference group [11].

## 4. Early Life Factors

Several early life factors have been associated with the development of T1DM later in life. One of the most well-studied is the mode of delivery. The cesarean section has been associated in many studies with an increased risk of developing T1DM. The proposed explanation is that by cesarean section, the fetus is not exposed to maternal flora, resulting in changes in its microbiome [35,36,37]. Therefore, the increase in the incidence of T1DM could be associated with the fact that worldwide caesarian section rates have tripled since 1990, increasing from 7% to 21% in 2021 [38].

Another early life factor whose relationship with the development of T1DM has attracted much attention is breastfeeding. Children who were never breastfed or who were breastfed for a shorter period of time have been found to be at increased risk of developing T1DM. On the contrary, two or more weeks of exclusive breastfeeding reduces the risk of developing T1DM later in life [39,40]. It is not clear whether the early introduction of cow milk has a negative effect or whether breastfeeding plays a protective role. In the Primary Prevention Study for Type 1 Diabetes in Children at Risk [41], genetically predisposed children were randomized into two groups, one of which received a hydrolyzed casein formula and the other formula based on cow’s milk. There was no difference in the occurrence of T1DM between the two groups, suggesting that cow’s milk does not have a detrimental effect on the triggering of the autoimmune process. Therefore, breastfeeding is more likely to reduce the risk of developing T1DM, through its effect on the gut microbiota and the infant’s immune system [42,43,44].

## 5. Microbial Exposure and the Hygiene Hypothesis

The hygiene hypothesis proposes that childhood exposure to microbes helps the immune system mature and develop, and thus in the absence of such exposure, the risk of autoimmune diseases increases [45,46]. In support of the hygiene hypothesis, there is a significant negative correlation between the incidence of T1DM by country and mortality from parasitic and infectious diseases, tuberculosis, respiratory infections and diarrheal diseases. In addition to being a marker of the quality of healthcare care provided, infectious disease mortality is also a marker of the total infectious burden [47]. Furthermore, maternal infections during pregnancy are associated with a lower risk of developing T1DM [48]. However, it should be noted that a reverse causality phenomenon in the association between T1DM and infectious diseases cannot be excluded, which means that the lower incidence of the disease in low-income countries could be related to lower survival rates of children because they receive poor quality health services.

Exposure to antibiotics is another factor that relates to the risk of developing T1DM. Exposure to a single antibiotic during the first year of life does not increase the probability of developing T1DM. However, exposure to two or more antibiotic treatments during the first year of life is a significant predictive factor for the subsequent development of T1DM [49,50].

## 6. Pollution

Exposure to increased concentrations of ozone in the air, both after birth and in utero, has been associated with an increased risk of developing T1DM [51,52,53]. Ozone can exert its diabetogenic effects by increasing oxidative stress [54,55], inflammation [56], and apoptosis [57]. Additionally, in animal studies, ozone has been reported to induce insulin resistance [58]. Exposure to particulate matter <10 μm (${\mathrm{PM}}_{10}$) and <2.5 μm (${\mathrm{PM}}_{2.5}$) in diameter of children before the age of 5 years has been recognized as an important prognostic factor for the development of T1DM. Particulate matter of this size can be inhaled and cause a number of adverse health effects. Furthermore, it is known to promote inflammation [59] and stimulate the release of interleukin 1β (IL-1β), which plays an important role in the destruction of β-cells in T1DM [60]. 

Other air pollutants with a strong correlation with the incidence of T1DM are nitrogen oxides that include nitrogen oxide (NO) and nitrogen dioxide (${\mathrm{NO}}_{2}$). Increased ambient levels of nitrogen oxides have a well-established dose-dependent association with IL-6 [59,60]. A large number of pollutants, in addition to those mentioned above, which can be found in the environment at elevated concentrations due to human activity, have been shown to increase the risk of developing T1DM in animal models, but studies in humans are scarce. These molecules include bisphenol A, organochlorine pollutants, mercury, and aluminum [61,62,63,64,65,66].

## 7. Ecological and Geographical Factors

The incidence of T1DM has recently been shown to increase as one moves closer to the sea [67]. There are several possible explanations for this phenomenon, including dietary habits, infection patterns, and the magnitude of pollution. Given that an increasing percentage of the world’s population lives close to the sea, the association mentioned above could help explain, at least partially, the rising incidence of T1DM. Furthermore, seasonal variation in the incidence of T1DM, with a peak observed during winter, and the higher prevalence of the disease in areas of high geographic latitude support the hypothesis that vitamin D plays a critical role in the pathophysiology of the disorder [68]. In the 0–14-year age group, the highest incidence has been observed in Northern Europe, Australia/New Zealand, and Northern America, whereas the lowest has been reported in Melanesia, Western Africa, and South America [69]. Although the mechanisms involved remain obscure, it has been suggested that vitamin D exerts immunomodulatory actions and has direct and indirect effects on β-cell apoptosis and autophagy and regulation of related genes, thus affecting insulin secretion and action [70].

Moreover, a lower prevalence of diabetes has been reported among people living at high altitude, compared to lowlanders. Although the exact underlying causes remain obscure, changes in the hormonal environment induced by altitude and hypoxia, different patterns of physical activity due to the demand to move and work in a rough terrain, limited access to health services, which could lead to underreporting of the actual prevalence and genetic determinants, have been suggested as potential explanations [68].

In support of the notion that the environment mediates the risk of the presentation of T1DM, several migrant studies demonstrated differences in the incidence of the disease between genetically “identical” populations living in different environments. In particular, these studies revealed that migrant populations acquire the T1DM risk profile of their new community [71,72,73]. In addition to climate conditions, cultural and ethnic factors may contribute to the magnitude of the change in the incidence of T1DM observed after migration [74]. Migration from developing and low-income to high-income countries has been a much more common occurrence in the last few decades than ever before in the history of humankind. Those migratory flows seem to expose an increasing number of people to an environment that triggers the onset of T1DM at a high rate.

## 8. Gut under the Microscope: The Role of the Microbiome

**Figure 1:**
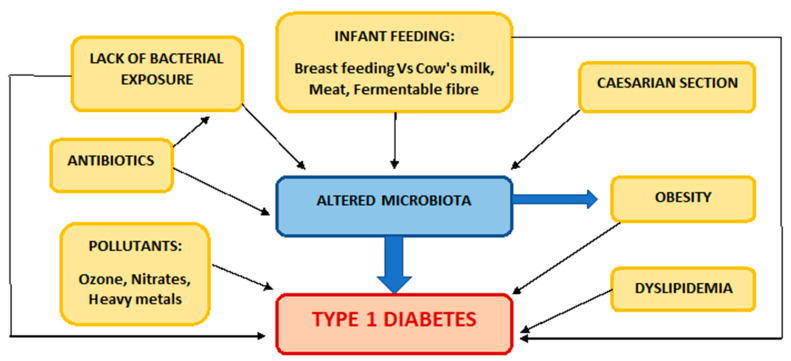
Factors related to the increasing incidence of T1DM.

As already discussed, several factors that have become more and more prevalent in recent decades seem to contribute to the increase in the incidence of T1DM (Figure 1). The authors believe that modulation of the microbiome is a key mechanism through which many environmental factors, including breastfeeding, mode of delivery, childhood diet, antibiotic use, and lack of microbial exposure, exert their effect on the risk of T1DM.

The gut microbiome consists of approximately 100 trillion bacteria belonging to 1000 different species that live in the gastrointestinal tract [75]. A disturbance in the equilibrium of the intestinal microbial community, generally described as ‘dysbiosis’, has been shown to be associated with the risk of developing various diseases, including autoimmune disorders, malignancies, and systemic infections [76]. Molecular mimicry has been recognized as one of the key mechanisms that lead to the development of T1DM. It has been suggested that ‘diabetogenic’ microbes in the gut share sequence similarity with beta cell epitopes, and through this mechanism, the over-representation of these microbes in the intestinal population can trigger or accelerate insulitis, which is the pathophysiological hallmark of the disease [77]. Similar microbiome abnormalities have also been described in other autoimmune diseases, such as systemic lupus erythematosus (SLE), where a lower *Firmicutes/Bacteroides* ratio has been observed compared to healthy controls [78]. It should be noted that in contrast to T1DM, which is mainly characterized by T cell activation, and in which autoantibodies represent only a marker of autoimmunity, in SLE, the role of B cells and autoantibodies is critical to promote tissue damage. This is indicative of the complexity of the mechanisms through which dysbiosis interferes with the immune system, and most of them have not been fully elucidated.

The integrity of the intestinal barrier is crucial to the separation of luminal antigens from internal organs. Disruption of this protective mechanism might lead to the leakage of antigens, such as toxins, food components, and inflammatory factors, into the systemic circulation and the subsequent activation and proliferation of CD8+ T cells that orchestrate β-cell damage. Several studies have shown that changes in the gut population of specific microbe species, including *Dialister invisus*, *Gemella sanguinis*, and *Bifidobacterium longum*, are associated with compromised intestinal mucosal integrity and also with an increased risk of T1DM [79,80].

Experimental data suggest that even a single course of macrolide treatment can induce lasting adverse effects on the topology and immunity of the microbial network [81]. In a mouse model of T1DM, *Lactobacillus johnsonii* administration resulted in down-regulation of oxidative response proteins in the intestinal mucosa, delaying in this way the onset of the disease [82]. Changes in the intestinal microbiota in mice with diabetes are associated with a loss of integrity of the intestinal barrier leading to the escape of antigens in the pancreatic lymph nodes, where they activate components of the immune system [83]. Changes in the microbiome profile induced by the antibiotic vancomycin have been reported to increase the risk of T1DM in non-obese diabetic (NOD) mice [84]. Different nutritional patterns can modify the risk of developing T1DM through changes in the composition of the gut microbiota. A gluten-free diet has been shown to protect against the disease by increasing the population of *Akkermansia* and reducing the population of *Bifidobacterium*, *Tannerella*, and *Barnesiella* species in NOD mice compared to a diet containing gluten [85]. Pectin, a dietary fiber, was shown to reduce the incidence of T1DM in NOD mice by increasing microbial species that produce short-chain fatty acids [86].

Studies in humans have produced equally promising results regarding an association between the microbiome and T1DM. Recent findings demonstrate that the microbiome composition of patients with T1DM differs significantly from that of normoglycemic individuals and is also correlated with clinical characteristics, such as quality of glycemic control, duration of diabetes, and burden of complications [87]. Fecal microbiota transplantation in patients with a recent (<6 weeks) T1DM diagnosis alleviated the sharp decrease in endogenous insulin production observed in autoimmune diabetes [88]. The microbiome profile of breastfed infants is characterized by the predominance of *Bifidobacteria* compared to children not breastfed [89], a fact that could explain, at least partially, the protective effect of breastfeeding against T1DM shown in several epidemiological studies [90]. Supplementation with probiotics during early life alleviates β-cell autoimmunity, as expressed by specific islet autoantibodies, among genetically predisposed individuals [91]. In a randomized, placebo-controlled trial that recruited children with T1DM, the administration of prebiotics resulted in significantly higher c-peptide levels and improved intestinal permeability after 3 months, compared to the placebo group [92]. However, this study did not show an improvement in glycemic control in the prebiotic group, which could be the result of the small sample size and the short intervention time [93]. It is worth noting that despite emerging data supporting the role of the microbiome in the pathogenesis of autoimmune diabetes, future studies must establish a causal association, as it is difficult to prove whether gut dysbiosis causes T1DM or vice versa.

Interestingly, several antidiabetic agents, such as metformin, have been shown to affect the composition of the microbiome [94]. However, whether insulin, which is the main treatment for patients with T1DM, can alleviate intestinal dysbiosis remains largely unexplored. Kihl et al. aimed to evaluate whether oral administration of porcine insulin to NOD mice can alter the gut microbiota [95]. The study failed to establish any significant effect of insulin on intestinal dysbiosis or incidence of T1DM. However, these negative findings might be due to oral insulin’s degradation during its passage through the intestine [75]; therefore, additional studies should be conducted with the use of subcutaneous insulin administration to shed light on the issue. Teplizumab, an immunomodulatory agent, has recently been approved to delay or prevent the onset of T1DM in high-risk individuals. Waldron-Lynch [96] et al. have shown that teplizumab administration increased interleukin-10-producing T cells that are considered protective against T1DM. These cells need to move into the intestine to exert their protective actions, providing some indirect evidence that the microbiome might be involved in the mechanism of action of the drug. However, this hypothesis deserves further evaluation in future studies.

A promising area for future research is whether the microbiome could serve as a marker for the prediction of the future risk of T1DM in genetically predisposed individuals or even as a marker of glycemic control and the risk of complications in patients with T1DM. Davis-Richardson et al. have demonstrated that changes in the intestinal microbiome become evident approximately 8 months before seroconversion in children with genetic risk of autoimmune diabetes [97]. Specifically, *Bacteroides dorei* and *Bacteroides vulgatus were* significantly higher in cases compared to controls prior to seroconversion. Future research will determine the cost-effectiveness and clinical implications of this highly promising screening method for T1DM. In general, although taxonomic and functional alterations in the intestinal microbiome have been well established in people with T1DM, the clinical utility of these observations, as well as their potential to serve as therapeutic targets, is still under investigation.

## 9. Conclusions

By understanding the exact mechanisms through which environmental factors play a role in the development of T1DM, we can shed light on the complex pathogenesis of the disease, identify new risk factors that are still unknown, and finally figure out what public health measures are required to halt the increase in new T1DM cases. Promoting a healthy lifestyle and diet, recommending alternatives to caesarian section and the prescription of antibiotics unless there is a medical indication, encouraging breastfeeding and reducing environmental pollution seem to be the most obvious but very challenging steps we can take to deal with the epidemic of T1DM. Quite unexpectedly, promoting pre-school daycare attendance could be another option to help deal with the rise of T1DM [48]. By increasing the exposure of children to microbes early in their lives, attendance at preschool day care, which went into a downward spiral during the COVID-19 era, has been inversely correlated with T1DM.

We seem to have many options to control the rise of T1DM, but most of them would require us to make drastic changes in our way of life. Thankfully, the ongoing COVID-19 pandemic proved that we can adopt such changes for the sake of public health.

## Data Availability

Not applicable.
